# In Situ-Based Gels for Nose to Brain Delivery for the Treatment of Neurological Diseases

**DOI:** 10.3390/pharmaceutics10020040

**Published:** 2018-03-30

**Authors:** Blessing Atim Aderibigbe

**Affiliations:** Department of Chemistry, University of Fort Hare, Alice Campus, Eastern Cape, Alice 5700, South Africa; baderibigbe@ufh.ac.za

**Keywords:** nose-to-brain delivery, neurological diseases, drug delivery systems, in situ-based gels, brain tumor, Alzheimer’s disease

## Abstract

In situ-based gel drug delivery systems that can bypass the blood-brain barrier, deliver the therapeutics to the desired site, reduce peripheral toxicity and control drug release kinetics have been developed. Some of the therapeutics used to treat neurological diseases suffer from poor bioavailability. Preclinical reports from several researchers have proven that the delivery of drugs to the brain via the nose-to-brain route using in situ gels holds great promise. However, safety issues on the toxicity of the nasal mucosa, transportation of the drugs to specific brain regions and determination of the required dose are factors that must be considered when designing these gels. This review will be focused on in situ-based gels that are used for the delivery of therapeutics via the nose-to-brain route, preclinical reports and challenges.

## 1. Introduction

Neurological diseases affect the peripheral and central nervous system. These include the peripheral nerves, spinal cord, brain, cranial nerves, nerve roots, neuromuscular junction, muscles, etc. [1]. Some examples of neurological disease are Parkinson’s disease, epilepsy, multiple sclerosis, Alzheimer’s disease, cerebrovascular diseases, brain tumors, etc. [1]. Neurological diseases affect millions of people worldwide. WHO (World Health Organization) estimated that 47.5 million people globally are suffering from dementia, and over 7.7 million new cases are reported every year [1]. The most common form of dementia is Alzheimer’s disease, which contributes to 70% of the cases. There are several factors that contribute to neurological disease such as genetics, physical injuries, infections, ageing, lifestyle, nutrition and environmental factors [2,3,4,5,6,7]. The treatment of neurological diseases includes the administration of the therapeutics topically, orally and intravenously and the use of device-based therapies such as deep brain stimulation, surgeries and rehabilitation [8]. Some approaches involve the direct delivery of the drug via injection into the brain, cerebrospinal fluid or intranasal delivery. Some of these techniques are unsafe, invasive, local and short lasting [9,10]. Another approach to the treatment of neurological diseases is the repair of the central nervous system, which involves the reconstruction of the damaged neural tissue. However, this approach is hampered by neurodegeneration [11]. The blood-brain barrier also acts as a barrier that inhibits the delivery of some therapeutic agents to the central nervous system and hinders drugs from passing through the endothelial capillaries to the brain [9]. In order to overcome the aforementioned limitations, gel-based drug delivery systems that can be administered via nose-to-brain routes have been developed. This approach is non-invasive, enhances drug absorption with less systemic adverse effects and bypasses BBB (blood-brain barrier). However, this approach has challenges such as the inability to know the accurate dose of drug to be administered and naso-mucosal irritation resulting from preservatives, additives and active ingredients added to the formulation that can cause loss of epithelial cell, shrinkage of the mucosal layer and loss of the ciliary layer [12]. Conditions such as allergy, flu, etc., can also interfere with the drug absorption [12]. This review will be focused on the gel-based drug delivery systems that are used for the delivery of therapeutics via the nose-to-brain route, preclinical reports and challenges.

## 2. Anatomy of the Nose

The nose is a primary organ that filters particles from the inspired air and provides immunologic defense. The inspired air comes in contact with the olfactory nerves and provides the sense of smell, which is closely associated with taste sensation [13]. The external opening of the nose is composed of nasal bone and cartilage. The nasal cavity extends from the external opening of the nose to the pharynx [14]. The internal part of the nose is composed of nasal septum that separates the nasal cavity into the left and right side [13]. The nasal cavity contains epithelium-covered bone. The roof of the nasal cavity contains a tissue known as the olfactory epithelium, which is useful for the sense of smell because it contains sensory cells. It protrudes from its surface and is embedded in mucus secreted by goblet cells in the epithelium. Olfactory sensory neurons are bipolar neurons at the surface of the epithelium in which their dendrites face the interior space of the nasal cavity. They transmit sensory signals to the brain [14]. Paranasal sinuses surround the nasal cavity and are characterized by hollow structures within the bones of the face. They are lined with epithelium and classified as maxillary, frontal, ethmoidal and sphenoidal sinuses [14]. The maxillary sinuses are the largest paranasal sinuses and flank the nasal cavity to the left and right. They are connected to the nasal canal via a tiny passage and allow the passage of air between them [14]. The frontal sinuses are separated from the nasal cavity by a thin layer of bone. They do not connect to the nasal canal [14]. The ethmoidal sinuses are also known as air cells. They differ in size and number in different people. The sphenoidal sinuses are round with varied sizes. The nasal cavity is divided into the vestibular, turbinate and olfactory regions [14]. The anterior part of the nose is the vestibular region. It is a very narrow part of the nasal cavity and is composed of vibrissae, which help in filtering particles sizes larger than 10 μm from the inhaled air [15]. The turbinate region is the principal site for the systematic absorption of drugs delivered intranasally. It is lined with a pseudostratified columnar epithelium and composed of cells such as ciliated, non-ciliated, basal and mucus-secreting cells [15]. Non-motile microvilli cover the ciliated and non-ciliated cells, and they are useful for enhancing the surface area and comprise a region where drug absorption occurs [15]. Motile cilia cover the cilia cells, and they are responsible for mucus transport, resulting in mucociliary drug clearance especially in the highly ciliated middle and posterior regions [15]. In the olfactory epithelium, the nerve cells project into the olfactory bulb of the brain providing a connection between the brain and the external environment, and this connection is useful for the transportation of drugs. Mucus covers the epithelium cells and traps unwanted particles. The mucus contains water, mucin, protein and salts. The proteins it contains are albumin, lactoferrin, immunoglobulin and lysozyme [15].

### 2.1. Mechanism of Nose-to-Brain Drug Delivery

**Figure 1:**
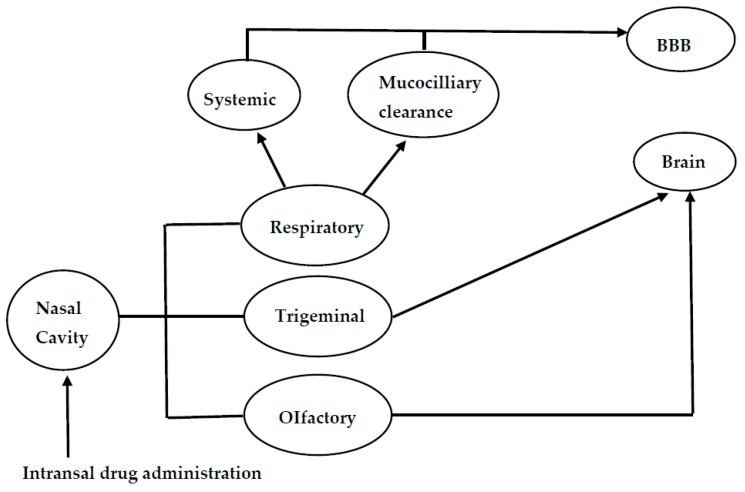
Schematic diagram of the mechanisms for nose-to-brain drug delivery.

The nasal mucosa has emerged as a useful target tissue for drug delivery when compared to oral drug administration resulting from its accessibility, high blood flow, large surface area, porous endothelial membrane and its ability to avoid hepatic first pass metabolism [16,17,18]. However, the exact mechanisms of drug delivery for nose-to-brain delivery is not well understood. The pathways involving the combination of the cerebrospinal fluid, vasculature and lymphatic system have been reported to be responsible for the transport of bioactive agents from the nasal cavity to the brain (Figure 1). However, the properties of the therapeutics and delivery system can result in one predominate pathway [19]. When the drug is deposited on the respiratory epithelium, the drug is absorbed into systematic circulation and then to the central nervous system. Drug deposited on the olfactory epithelia is transported to the central nervous system through paracellular or transcellular transport via olfactory neurons or olfactory epithelial cells. Another route for the transportation of drug from the nose to the brain is via the trigeminal nerves. The olfactory and trigeminal nerve system connects the brain and nasal cavity. These nerve systems are externally-accessible points of the brain, which can be exploited to bypass the BBB for direct nose-to-brain drug delivery [20]. The major route of intranasal delivery is the olfactory neural pathway in which the drug is transported via the olfactory nerve axons, to the olfactory bulbs and then into the brain. The aforementioned pathway of drug delivery across the olfactory epithelium can be further classified as: (i) a transcellular pathway, which involves receptor-mediated endocytosis, fluid phase endocytosis, or passive diffusion; (ii) a paracellular pathway via tight junctions between the sustentacular cells of the olfactory epithelium and olfactory neurons; (iii) the olfactory nerve pathway in which drug uptake is by endocytotic or pinocytotic mechanisms and transportation of the drug to the olfactory bulb is via intracellular axons [20].

### 2.2. Anatomical Structures Involved in Nose-to-Brain Transport

**Figure 2:**
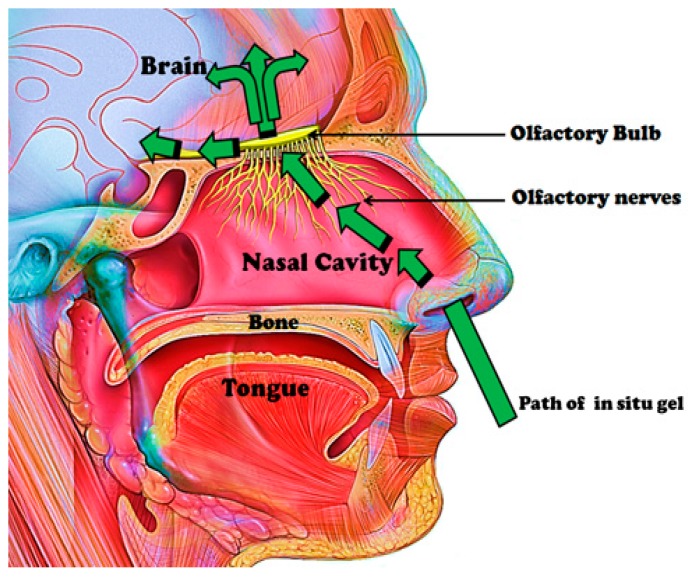
Anatomical structures involved in nose-to-brain transport.

Some anatomical structures are involved in nose-to-brain transport (Figure 2). The nasal mucus pH is within the range of 5.5–6.5, and it is moved by the cilia on the respiratory mucosa. The amount of mucus in the nasal mucosa can influence the drug absorption [21]. It is the first barrier that drugs administered intranasally must cross before travelling between cells either paracellularly or transcellularly [22]. Drug administered is transported between the epithelial cells, and these cells can be close via several junctions referred to as tight junctions, desmosomes, adhering junctions and gap junctions [22,23]. The unaltered state of these junctions influences the paracellular transport. Some therapeutics can open these junctions, thereby enhancing nose-to-brain delivery, and this route is rapid [22]. It is important to mention that the size of the therapeutics influences the mechanism of drug delivery. Therapeutics larger than 20 nm are reported to be transported transcellularly. Therapeutics smaller than 200 nm and in the range of 200–1000 nm undergo caveolae-mediated endocytosis and clathrin-mediated endocytosis, respectively. Transcellular drug transport is slow, and the bioactive agents are transported to the olfactory receptor neuron and undergo intraneuronal transport to the olfactory bulb [22,24].

Bioactive agents can be transported via the olfactory nerve fibers of the olfactory bulb to the CNS (Central Nervous System). The olfactory bulbs projects to the different regions of the brain such as the hypothalamus, olfactory tract, amygdala, entorhinal cortex, anterior olfactory nucleus, etc. Intra- and peri-neural transport occurs via these projections upon intranasal drug administration [22]. The respiratory region in the nasal cavity is supplied with nerves by projection of the trigeminal nerves, which are composed of branches such as the ophthalmic, maxillary and mandibular nerves. These branches enter the brain at the cribriform plate and the lacerated foramen, thereby producing entrance into the rostral and caudal regions of the brain [22]. The olfactory region’s vascularization is another feature useful in nose-to-brain transport, and it originates from small branches of the ophthalmic artery. Drug administered intranasally enters the systemic circulation via this vascularization, thereby bypassing the BBB to the brain. This is applicable to small and lipophilic drugs. Drugs can travel via channels between the outermost layer of blood vessels and the basement membrane of surrounding tissue by the perivascular pathway [22,25]. This pathway is driven by bulk flow, diffusion and arterial pulsation, resulting in rapid drug distribution in the CNS [25].

## 3. Neurological Diseases and Challenges in Treatment

The treatment of most neurological diseases is challenging due to the blood-brain barrier, their association with a number of genes, the overlap of disease-associated genes, drug side effects with minimal effects on the disease progression and mechanisms and biomarkers behind neurological diseases not being well understood [26,27,28].

Alzheimer’s disease is a neurological disorder in which the brain nerve cells are destroyed, resulting in dementia. It is characterized by intracellular neurofibrillary tangles, insoluble β-amyloid (Aβ) peptides/senile plaques and the loss of different neurons in the basal forebrain amygdala, cortical and hippocampus region of the brain [17]. The risk factors for Alzheimer’s disease are decreased availability of oxygen resulting in cell death [29], head injury [30], vitamin D deficiency [31] and a high concentration of copper and homocysteine levels resulting in damage to the neurons [32]. The current approach in the management of Alzheimer’s disease is symptomatic, which involves the counterbalance of neurotransmitter disturbance of the disease by administration of cholinesterase inhibitors [17]. The United States Food and Drug Administration (FDA) has approved five drugs for the management of Alzheimer’s disease, which include donepezil, galantamine, rivastigmine, memantine and the combination of donepezil with memantine. Donepezil, galantamine and rivastigmine are referred to as cholinesterase inhibitors, and they treat symptoms associated with memory, language, etc. They act by maintaining the levels of acetylcholine, which then compensates for the loss of the functioning brain cells [33]. Memantine regulates the activity of glutamate, an important neurotransmitter, which is important in learning and memory. It also protects the cells against excess glutamate by partially blocking MNDA (N-methyl-D-aspartate) receptors [33]. The combination of donepezil with memantine is used to treat moderate to severe stages of the disease. These drugs suffer from some side effects (Table 1) [33].

Multiple sclerosis is an autoimmune disease, and it affects the central nervous system, resulting in the damage of the axon, demyelination and loss of neurological functions [34]. Factors that are believed to contribute to the disease are common childhood infections, low level of exposure to sunlight (vitamin D deficiency), smoking and genetic factors [34]. The loss of axons is reported to be initiated by complex inflammatory processes [34,35]. There is presently no cure for multiple sclerosis. Two major strategies that have been reported to be potential routes to the treatment of the disorder are to hinder the immune system from causing damage that results in the clinical manifestations of the disease and to establish mechanisms that will result in the CNS being resistant to the deleterious effects of the immune response, known as neuroprotection [36]. The treatment approach focuses on treating the symptoms, slowing the progressive forms of the disease and speedy recovery from attacks [34]. Drugs used to treat the disorder are known as immunomodulatory agents because they modify the immune response, thereby reducing the deleterious effects mediated by the immune system. However, the mechanisms of these drugs are not well understood [36]. Some of the drugs used include beta interferons, fingolimod, glatiramer acetate, teriflunomide, dimethyl fumarate, mitoxantrone, natalizumab, etc. These drugs suffer from severe side effects, resulting in poor patient compliance such as irritation at the site of injection, influenza-like symptoms, chest tightness, heart palpitations and breathlessness, heart failure, leukemia, etc. [34,37]. Other neurological diseases are schizophrenia, epilepsy, Parkinson syndrome, brain tumor, etc.

Schizophrenia is a chronic mental health disorder, which is characterized by delusions, hallucinations, disorganized behavior, etc. [38]. It is caused by an excess or a deficiency of neurotransmitters, which include glutamate, dopamine and serotonin. Dopaminergic pathways that attribute to the disease are the nigrostriatal pathway in which low dopamine levels affect the extrapyramidal system, resulting in motor symptoms; excess dopamine in the mesolimbic pathway that extends from the ventral tegmental area to limbic areas; low mesocortical dopamine levels in the mesocortical pathway; a decrease in the tuberoinfundibular dopamine in the tuberoinfundibular resulting in elevated levels of prolactin, amenorrhea and reduced libido [38]. Other factors that are genetic and environmental also contribute to the disease. The condition is managed using antipsychotic drugs, which suffer from adverse effects such as diabetes mellitus, weight gain and hyperlipidemia, which increase the risk of cardiovascular mortality (Table 1) [38,39]. Other complications associated with the drugs are dystonia, cataracts, sexual dysfunction, etc. [38].

Epilepsy is a non-contagious and chronic disorder of the brain characterized by recurrent seizures caused by excess electrical discharges in a group of brain cells [40]. Factors that contribute to the disease are malaria, birth-related injuries, severe head injury, meningitis, encephalitis, neurocysticercosis, brain tumor, etc. [40]. Some of the drugs used for the treatment of epilepsy exhibit adverse effects such as aplastic anemia, hepatitis, allergic rashes, etc. [40]. The main limitation of the drugs used to treat epilepsy in some patients is pharmacoresistance resulting from mechanisms that are disease related, genetics and drug related. The disease-related mechanism is responsible for the alterations of pharmacological targets of antiepileptic drugs in the brains of pharmacoresistant patients leading to the failure of antiepileptic drugs to block excitatory sodium or calcium currents. The genetic alterations result from drug efflux transporters leading to poor seizure control, and the drug-related mechanism is responsible for the reduced efficacy of antiepileptic drugs [41,42].

Parkinson syndrome is characterized by Lewy bodies containing aggregations of the protein alpha-synuclein and the loss of pigmented melanin-containing neurons in the midbrain [43]. The loss of pigmented melanin-containing neurons in the midbrain reveals neurodegeneration of dopaminergic neurons in the substantia nigra characterized by dopamine deficit in the striatum [43].

The treatment of Parkinson’s disease is classified as a symptomatic and neuroprotective therapy. Presently, there is no proven neuroprotective therapy for the treatment of the disease. In cases of severe Parkinson’s syndrome disease, when the medication is longer effective, brain surgery, which involves deep brain stimulation, is performed so as to manage the motor symptoms [44,45]. The drugs that are used to treat the disease are classified as dopamine precursors, dopamine agonists, monoamine oxidase B inhibitors and anticholinergics [44,45]. These classes of drugs suffer from some side effects, as illustrated in Table 1.

A brain tumor is characterized by an abnormal growth of tissue in the brain or central spine, disrupting the function of the brain, which can be either cancerous or non-cancerous [46]. Tumors are classified based on where the cells originated. They are classified as benign when they originate from cells within the brain and do not spread into other tissue; malignant brain tumors that spread into other tissues and grow rapidly, thus invading the surrounding brain tissue; primary tumors that start in cells of the brain and can spread to other parts of the brain or to the spine; metastatic brain tumors that begin in another part of the body and then spread to the brain [46]. The risk factors are viral infection, chemicals, ionizing radiation and genetic manipulation [47]. Brain tumors are difficult to treat. Some of the drugs used for the treatment of brain cancer are temozolomide, lomustine, bevacizumab, carmustine wafer, etc.

## 4. Nasal Delivery for the Treatment of Neurological Diseases Such as Alzheimer’s Diseases, Parkinson’s Diseases, Epilepsy, etc.

Gel-based drug delivery systems, which can be used to administer drugs intranasally, have been developed for the management of neurological diseases. These systems are biodegradable, biocompatible, can deliver the drugs to the brain, bypass the blood-brain barrier and are potential therapeutics for the treatment of neurological disorder. The design of these systems, the concentration of polymers, pore sizes and the rate of degradation influence their efficacy.

### 4.1. Hydrogels

Hydrogels are hydrophilic, cross-linked networks of water-soluble polymers, which can retain a large amount of water [48,49]. They can be formulated in various physical forms such as slabs, films, in situ hydrogels, nanogels, microparticles, nanoparticles, etc. [48]. They can be easily modified with selected functional groups and exhibit pores with sizes that can be controlled by the density of crosslinking. Their porosity is useful for the loading and the release of drugs at a rate that is influenced by the diffusion coefficient of macromolecules through the hydrogel network [48,49]. Hydrogels are highly biocompatible, which is attributed to their high water content and their physiochemical properties, which are similar to the native extracellular matrix [48,49]. The degree of biodegradability of hydrogels is designed into the hydrogel via selected pathways such as environmental [49]. They can also be deformed to the shape of the surface to which they are to be delivered [49]. They are sensitive to external stimuli such as temperature, pH and magnetic field. They are prepared by different methods, and the method of preparation influences their pore size, rate of degradation, mechanical strength and drug release mechanism. Due to their unique physicochemical properties, they have been designed for nose-to-brain delivery.

#### 4.1.1. In Situ Gels

In situ-based gels are systems that exhibit sol-to-gel transition at the site where they are administered into the body. They are liquid when administered and undergo a sol-to-gel transition induced by external stimuli such as temperature, pH, ion change and magnetic field or in the biological environment [48,50]. They exhibit good properties, which make them useful for drug delivery such as: they are highly compatible with a range of drugs, which are soluble, insoluble, low and high molecular weight drugs; they are less invasive and can be used to obtain high drug concentrations at the desired site of action with reduced systemic side effects; biocompatibility; biodegradable and exhibit sustained drug release over an extended period, thereby enhancing patient compliance [51]. The aforementioned properties make them useful for nose-to-brain delivery.

##### In Situ-Based Gels for the Delivery of Anti-Parkinson Drugs

**Figure 3:**
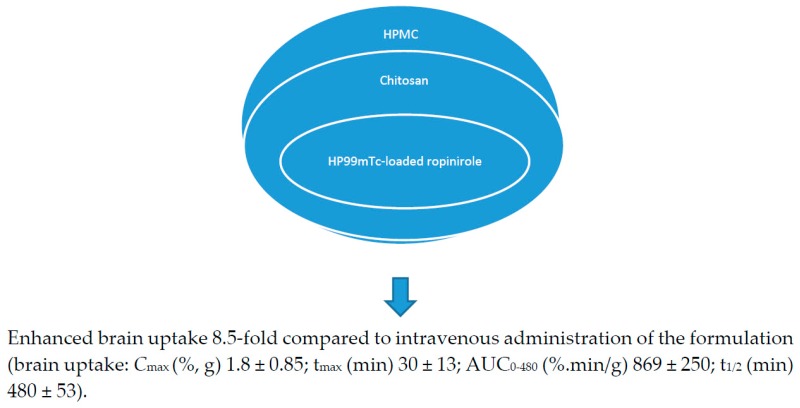
Chitosan-based in situ gels for the delivery of ropinirole.

Anti-Parkinson drugs such as levodopa are used for the treatment of Parkinson’s disease, and its use is limited by its poor bioavailability, which is characterized by its low brain uptake. Its poor bioavailability is attributed to the irregular gastrointestinal metabolism of the drug before it attaches to the l-amino acid carrier that transports the drug actively through the duodenum where it enters the bloodstream [52,53,54,55]. Sharma et al. incorporated chitosan nanoparticles loaded with levodopa prepared by ionic gelation technique using sodium TPP (1 mg/mL) onto a thermo-reversible gel prepared from Pluronic PF127 (Poloxamer 407) [56] (Table 2). The formulations were characterized, followed by in vitro drug release studies, which revealed that the formulation obeyed the Hixson-Crowell model (drug release by dissolution with changes in the surface area and diameter of the formulation). The addition of polycations enhanced the drug absorption of the formulation on the nasal mucosa by opening the junctions between epithelial cells and delaying mucociliary clearance. In vivo studies on Swiss albino rat models further showed that intranasal administration of the chitosan nanoparticles resulted in an enhanced brain uptake of the drug when compared to the gel formulation, suggesting that the viscosity of the gel reduced the brain uptake of the drug [56]. Lungare et al. developed in situ thermoresponsive-based gels by the cold method using Pluronic F127 (Poloxamer 407) as a thermoreversible polymer and carboxymethylcellulose as a mucoadhesive polymer [57]. It was loaded with amantadine, an anti-Parkinsonian drug. Increasing the mucoadhesive polymer resulted in an increased gelation temperature, and increasing amantadine reduced the gelation. A concentration of 16% of Pluronic F127 was found to be suitable for the sol-to-gel transition of the formulation at ambient nasal temperatures. These systems are potential therapeutics for the treatment of Parkinson disease [57]. The formulation was stable, which was evidenced by the repeatable drug release profiles of the Fickian mechanism (a transport process in which the polymer relaxation time is greater than the solvent diffusion time), followed by an anomalous drug release mechanism (a combination of diffusion and erosion controlled drug release) after storage of the formulation at 4 °C for eight weeks. There was no significant cellular toxicity to the human nasal epithelial cells up to 4 mg/mL and up to 1 mM. The % drug release from the formulation was in a range of 43–44% in vitro [57,58]. Khan et al. reported mucoadhesive in situ gel formulation prepared from chitosan and hydroxyl propyl methyl cellulose for intranasal delivery of ropinirole to the brain [59]. In vivo brain uptake of ropinirole in albino rats following intranasal administration of 99mTc (Technetium 99m)-ropinirole-loaded gel AUC (area under the curve) (0–480 min) was 8.5-times when compared to the intravenous administration [59] (Figure 3). Ravi et al. prepared thermosensitive gel for intranasal delivery of rasagiline mesylate, a drug for the treatment of Parkinson’s disease [60]. The gels were prepared from a combination of poloxamer 407 and poloxamer 188 in a 1:1 ratio with mucoadhesive polymers, namely: carbopol 934 P and chitosan. In vivo performance of the formulation in New Zealand white rabbits suggested that the intranasal administration of the formulation exhibited a better drug bioavailability of six-fold higher than the oral solution. Nasal mucosal integrity studies indicated maintained integrity of the nasal mucosa of rats after chronic administration of the formulation. The brain uptake of the formulation was significantly (*p* < 0.01) high when compared to the drug solution. Rasagiline mesylate’s poor bioavailability is attributed to its rapid absorption and first-pass metabolism, which is overcome when administered intranasally, resulting in extended residence and contact time with nasal epithelium and enhanced drug absorption from the nasal cavity. High *C*_max_ revealed the rapid absorption of the drug, and the high AUC_0–∞_ values suggested complete absorption of the drug from the gel formulations [60]. Rao et al. prepared thermoreversible nasal gels by the cold method from Pluronic F-127 and hydroxy methyl propyl cellulose, and ropinirole, an anti-Parkinson drug with poor oral bioavailability, was loaded onto the gel [61]. Formulations exhibited gelation at the nasal temperature, and the time of gelation was less than the mucociliary clearance time. The nasal residence time of the formulation was influenced by the mucoadhesion and enhanced strength of the gel. The formulations’ ex vivo drug release was 56–100% over a period of 5 h. Histological study of sheep nasal mucosa revealed that the gel had a protective effect when compared to the free drug, which was characterized by cellular damage. The brain uptake of the drug after nasal administration was five-fold when compared to the administration of the formulation intravenously, revealing the system as a potential delivery system for anti-Parkinsonian drugs. The drug delivery from the formulation to the brain was via the olfactory nerves [61].

##### In Situ Gels for the Delivery of Anti-Migraine Drug

Sumatriptan succinate is used for the treatment of migraine. It suffers from poor bioavailability, which is associated with its low retention time in the nasal cavity and its low delivery to the brain via the olfactory pathway. The permeation of sumatriptan across the brain-blood barrier is very poor. Galgatte et al. prepared in situ gel in a simulated nasal fluid using deacetylated gellan gum as the gelling agent [62]. The strength of the gel and the rate of drug release from the gel was influenced by the concentration of gellan gum in the formulation. The strength of the gel increased with the increase in the concentration of gellan gum, while the rate of drug release decreased with the increase in the concentration of the gellan gum. The release mechanism of the drug from the gel followed a Fickian release model, revealing an erosion diffusion mechanism. Ex vivo permeation was studied using sheep olfactory nasal mucosa with a thickness of 0.6 mm. Its permeation showed a release of 93% of the drug over a period of 300 min. The microscopic structure of the mucosa did not reveal cell necrosis after the application of the formulation. The interaction between the drug and the excipients was not significant, suggesting the absence of local irritation to the nasal mucosa. The formulation was stable at a room temperature of 25 °C and at 4 °C. The concentration of sumatriptan in the plasma and the brain tissues was higher by nasal in situ gel compared to the oral aqueous solution. In vivo studies showed that AUC was higher in the plasma and the brain for the formulation administered by the nasal route when compared to oral administration. The AUC of sumatriptan in brain tissues was 1.44-times higher when compared to the AUC in plasma when the formulation was administered intranasally. The results revealed that the permeation of drug molecules across the nasal mucosa to the brain was via the olfactory pathways (Table 2) [62].

##### In Situ Hydrogels for the Delivery of Anti-Alzheimer’s Drug

Selected anti-Alzheimer’s drugs have been loaded onto in situ gels, resulting in an enhanced brain uptake of the drug in vivo. Tao et al. prepared gellan gum-based in situ gel loaded with huperzine A. The gel was prepared by the precipitation method (Table 2) [63]. Huperzine A uptake by the rat brain tissues after intranasal administration indicated that the AUC (0–>6 h) value in plasma obtained after nasal administration was 0.94 compared to the intravenous administration. The AUC (0–>6 h) of cerebrospinal fluid after nasal administration was 1.3- and 2.- times compared to intravenous and intragastric administration. The in situ gel significantly increased the distributions of huperzine A in the rat brain tissues, especially in the cerebrum and hippocampus [63]. Chen et al. loaded curcumin onto thermosensitive hydrogel for good brain targeting efficiency. The gels were prepared from pluronic F127 and poloxamer 188 [64]. The gels exhibited shorter gelation time, extended mucociliary transport time and prolonged curcumin retention in the rat nasal cavity at body temperature. The curcumin release mechanism from the gel was diffusion and dissolution controlled, respectively, when the dialysis membrane method and membraneless methods were employed. The nasal mucosal integrity was maintained over a period of 14 days after application of the formulation. The drug-targeting efficiencies for curcumin in the cerebrum, cerebellum, hippocampus and olfactory bulb after intranasal administration of the formulation were 1.82-, 2.05-, 2.07- and 1.51-times, respectively, when compared to the drug-targeting efficiencies of the drug after intravenous administration of the curcumin solution. The gel increased the drug uptake and distribution in the rat brain tissue, which was significant in the cerebellum and hippocampus [64]. Wang et al. prepared thermoreversible in situ nasal gel by the cold method from poloxamers (P407, P188) and the hydroxypropyl methylcellulose for the delivery of geniposide [65]. Borneol was employed as a permeation enhancer. The drug content of the formulation was in the range of 97–100%; the gel strength and mucoadhesive strength of the formulation was in the range of 25–50 s and 4000–6000 dyn/cm^2^, respectively. The in vitro release mechanism of geniposide was zero-order, and the ex vivo release mechanism of geniposide obeyed the Weibull model (a mechanism in which the amount of drug release is a function of time), suggesting that the release of geniposide is influenced by gel corrosion and that the permeation of geniposide is time dependent [65]. The finding revealed the potential of the formulations for the treatment of neurological diseases. Salatin et al. developed in situ gel from poloxamer 407^®^ for the delivery of rivastigmine hydrogen tartrate in poly(lactic-*co*-glycolic acid) nanoparticles [66] A high drug permeation through the sheep nasal mucosa was observed when compared to the free drug. The formulation was stable; embedding the drug in nanoparticles enhanced the permeability; and the drug release from the formulation was sustained. The cellular uptake of the drug from the formulation was time dependent and was cytocompatible on A549 cells [66]. Abouhussein et al. also investigated brain delivery of rivastigmine tartrate via mucoadhesive thermosensitive in situ gel intranasally [67]. The mucoadhesive in situ gel was developed from pluronic F127, HPMC (hydroxypropyl methylcellulose), chitosan, carbopol 934 and NaCMC (sodium carboxymethyl cellulose). In vivo pharmacokinetic and biodistribution studies using the radiolabeling approach in normal albino mice revealed 84% intranasal permeation with a good distribution to the brain (0.54% ID/g) when compared to intravenous administration. Intravenous administration of the drug solution resulted in a high drug distribution to liver and kidney compared to administration of the formulation intranasally. These findings suggested that intranasal drug administration reduced drug systemic distribution to different organs, thus resulting in enhanced drug targeting to the brain, hence overcoming side effects [67]. The *C*_max_ and AUC of brain concentrations of (0.58% radioactivity/gram) and (84.7% radioactivity.min/gram) were significantly high. The AUC_0-∞_ was five-fold greater when administered intranasally when compared to the free drug solution administered intravenously, suggesting drug transport to the brain was via the olfactory route. The mucoadhesive nature of the CP 934 polymer used hindered the normal mucociliary clearance of the formulation [67].

##### In Situ Gels for the Delivery of Anti-Depressant Drug

Doxepin is an anti-depressant drug. Naik and Nair reported thermoreversible gels prepared using chitosan and glycerophosphate or poly(ethylene) glycol for the delivery of doxepin to the brain via intranasal administration. In vivo studies in Swiss albino mice showed a good increase in activity count and a decrease in immobility time, suggesting good antidepressant activity (Table 2) [68]. The drug solution caused a significant damage to the nasal mucosal tissues, which was characterized by glandular hyperplasia and severe epithelial hyperplasia. However, the administration of the gel formulation resulted in mild side effects, which include mild swelling of glands, and there was no symptom of sluffing of the mucosal epithelium, which was observed in mice in which the drug solution was administered. The rate of permeation of doxepin from the gel matrix was influenced by its release profile from the matrix. The drug from the formulation prepared from chitosan, glycerophosphate or poly(ethylene) glycol permeated at a lower rate when compared to the formulation prepared from chitosan and glycerophosphate [68]. Fatouh et al. prepared in situ gel loaded with agomelatine, an antidepressant drug [69]. The drug release from the gel formulations was between 8.9–21% when compared to the drug release from the drug solution and solid lipid nanoparticles, which was 89% and 35%, respectively. The in situ gel exhibited significantly higher *C*_max_, AUC (0–360 min) and absolute bioavailability of 247 ng/mL, 6677 ng·min/mL and 38%, respectively, when compared to oral suspension of Valdoxan® (21 ng/mL, 2828 ng·min/mL and 16%, respectively (*p* < .0001)). The increase in *C*_max_, AUC 0–360 min and absolute bioavailability of the gel is due to the increased amount of the drug that bypasses the hepatic metabolism to reach the systemic circulation; the lipid nature of the particles, which permits their partition into the lipid bilayer of the nasal epithelial cell membrane and to penetrate the cells; and the co-surfactants used enhanced the permeability of the membrane structure by opening of the tight junctions between epithelial cells. Drug concentrations in the brain of rats after administration of gel formulation revealed a significantly high *C*_max_ and AUC (0–360 min) (148 ng/mL and 6570 ng min/mL, respectively) compared to the oral suspension (61 ng/mL and 1710 ng·min/mL, respectively) (*p* < .0001). [69]. Pathan et al. reported ion sensitive in situ nasal gel loaded with fluoxetine hydrochloride for brain delivery. The gel was prepared from gellan gum and HPMC (hydroxypropyl methylcellulose) [70]. Ex/in vivo permeation studies revealed that increasing the concentration of gellan gum from 0.2–0.6% and HPMC from 0.1 to 0.2% decreased the rate of drug release. The percentage of drug permeation over a period of 240 min from all formulations was between 75% and 94%. The integrity of the epithelial cell in the nasal mucosa treated with the formulation was maintained, indicating the non-toxic nature of the formulations. In vivo study further revealed that the administration of the formulation reduced the total immobility period and increased climbing and swimming behavior [70]. Kaur et al. studied the brain delivery of tramadol hydrochloride using mucoadhesive thermo-reversible gel [71]. The gels were prepared from chitosan nanoparticles by the ionic gelation method followed by the addition of the nanoparticles in Pluronic and HPMC-based mucoadhesive thermo-reversible gel. The formulation significantly hindered forced swim-induced depression by involving anti-oxidant-like effects, significantly increased locomotor activity and body weight of the rat model in vivo. The nanoparticles further enhanced the delivery of drug to the brain [71]. Pathan and More developed thermoreversible gel loaded with nortriptyline hydrochloride for intranasal administration [72]. An increase in the concentration of poloxamer 188 and HPMC increased the viscosity and mucoadhesive strength and decreased the gelation temperature and drug percentage permeation. The formulation with 3.6% poloxamer and 0.04% HPMC exhibited a 98% drug release through sheep nasal mucosa. The formulation was stable over a period of three months, and the results obtained indicated that the formulation is a potential therapeutic for the treatment of depression [72]. Haque et al. prepared venlafaxine-loaded alginate nanoparticles for the treatment of depression by intranasal administration [73]. Pharmacodynamic studies of the formulation for the antidepressant activity in vivo in adult Wistar rats showed improved swimming and climbing and reduced immobility (*p* < 0.01) when compared to the depressed group. The formulation enhanced the drug concentration in the brain, which is attributed to factors such as: increased absorption time, reduced nasal mucociliary clearance, increased permeation across nasal mucosa and modulation of P-gp efflux transporters present on BBB [73]. The brain/blood ratios of the formulation administered intranasally, drug solution administered intravenously and drug solution administered intranasally were 0.11, 0.03 and 0.07, respectively, at 30 min, which revealed the potential of the formulation for nose-to-brain delivery. The brain concentration of drug after intranasal administration of the drug was 743 ng/mL, t_max_ 60 min, and it was significantly higher (*p* < 0.05) than the drug solution administered intravenously (382.91 ng/mL; t_max_ 30 min) and drug solution administered intranasally (397 ng/mL; t_max_ 60 min) [73].

##### In Situ Gels for the Delivery of Anti-Schizophrenia Drug

Sherje et al. developed in situ gels from carbopol 934 and hydroxypropyl methyl cellulose loaded with paliperidone for the treatment of schizophrenia (Table 2) [74]. The formulation exhibited good mucoadhesion with sustained drug release. The nasal mucosa architecture remained unaffected after treatment with the formulation. The formulation exhibited a high rate of drug permeation through sheep nasal mucosa, which revealed the role of HP-β-CD (2-Hydroxypropyl)-β-cyclodextrin) as a nasal absorption enhancer [74].

## 5. Challenges and Future Perspective

The treatment of neurological diseases is challenging because of the number of genes associated, the progressive nature of the disease and the insufficient knowledge of the mechanisms and biomarkers associated with these diseases. Due to the aforementioned factors, the approaches used to manage these diseases include symptomatic and neuroprotective therapy. Most of the drugs used to manage these diseases suffer from severe side effects, and the drugs are useful at a selected stage of the disease. In order to overcome the severe side effects associated with some of these drugs and to improve the brain uptake, some researchers have studied some of these drugs in vivo when loaded together with nanoparticles onto in situ gels and administered intranasally. The delivery of drugs to the brain is a challenging and complex approach that requires collaborative research from different scientists from various fields ranging from the biomedical field to physical and material scientists. The most challenging task is the design of therapeutics that can target the right subset of diseased neurons without affecting the healthy neurons [75]. The BBB is permeable to lipophilic molecules with a molecular weight that is less than 600 Dalton, and the low permeability of BBB is also associated with low levels of pinocytosis and tight junctions, which are important for maintaining homeostasis in the central nervous system [22,76,77]. However, therapeutics administered intranasally can bypass the BBB.

The advantages of the nose-to-brain route for the delivery of therapeutics include the lower risk of systemic side effects and renal clearing, non-invasiveness, high patient compliance and rapid onset of action of the drug [22]. However, the exact mechanisms in the nose-to-brain route of drug delivery are not yet fully understood. The various preclinical studies have demonstrated the efficacy of in situ gels as a potential therapeutic platform for the intranasal administration of bioactive agents for the treatment of neurological disease. However, some of the challenges with intranasal drug delivery are enzymatic degradation of the drug molecule in the lumen of the nasal cavity, low membrane permeability and mucociliary clearance. The aforementioned challenges are overcome by the addition of absorption enhancers and bioadhesive excipients in the formulation, thereby enhancing the efficacy of the formulations in vivo. The development of in situ gels used in combination with bioactive agents and nanoparticles has increased drug delivery performance to brain tissue using novel targeting moieties in vivo [78]. Most of the designed gels have undergone only preclinical studies, and there is a need for these developed gels to reach clinical trials. Translating animal data to humans must be handled carefully because of factors such as the distinct differences in the anatomy of animals and humans. The nasal passage of humans is not as complicated as the one in rodents, which is characterized by a large surface to volume ratio, and primates are oronasal breathers, whereas rodents are obligatory nasal breathers. The nasal passage in a rat is more complex than in humans [22]. There is a need for more studies in order to understand the mechanism of the delivery of drug to the brain in neurological diseases after intranasal administration. There is also the need to develop new excipients that can enhance the drug bioavailability. Extensive toxicodynamic studies of excipients, nanoparticles and polymers used in the preparation of the gels are lacking. From the research findings obtained so far, it is very likely that in the near future, drugs for the treatment of neurological diseases in the form of nasal in situ gel formulations will reach the clinical stage.

## Figures and Tables

**Table 1:** Some drugs used for the treatment of selected neurological diseases.

Drugs	Application	Route of Administration	Side Effects
Donepezil	All stages of Alzheimer’s disease	Oral	Nausea, increased bowel movements, loss of appetite
Galantamine	Mild to moderate Alzheimer’s disease	Oral	Nausea, increased bowel movements, loss of appetite
Rivastigmine	Mild to moderate Alzheimer’s disease	Orally and transdermally	Dizziness, constipation, headache
Memantine	Moderate to severe Alzheimer’s disease	Oral	Dizziness, constipation, headache
Donepezil and memantine	Moderate to severe Alzheimer’s disease	Oral	Nausea, increased bowel movements, loss of appetite, dizziness, constipation, headache.
Beta interferon	Relapsing-remitting multiple sclerosis	Subcutaneous and intramuscular	Myalgia, headache, anemia, nausea
Glatiramer acetate	First clinical episode of multiple sclerosis	Subcutaneous	Nausea, vomiting, body ache
Fingolimod	Relapsing form of multiple sclerosis	Oral	Headache, diarrhea, cough
Teriflunomide	Relapsing form of multiple sclerosis	Oral	Liver problems, influenza, nausea, diarrhea
Dimethyl fumarate	Relapsing form of multiple sclerosis	Oral	Burning feeling of the skin, itching, nausea, abdominal pain, vomiting
Mitoxantrone	Worsening relapsing remitting multiple sclerosis	Intravenous	Nausea, diarrhea, constipation, flu-like symptoms, cardiac toxicity and risk of leukemia
Natalizumab	Relapsing progressive form of multiple sclerosis	Intravenous	Headache, stomach pain, diarrhea, depression
Quetiapine	To treat depression associated with schizophrenia	Oral	Risk of diabetes, weight gain, constipation, dizziness
Risperidone	Schizophrenia	Oral, intramuscular	Risk of diabetes, weight gain, constipation, dizziness
Paliperidone	Schizophrenia	Oral, intramuscular	Risk of diabetes, weight gain, headache.
Aripiprazole	Schizophrenia	Oral, intramuscular	Dizziness, nausea, vomiting, sedation
Asenapine	Schizophrenia	Oral	Weight gain, risk of diabetes, sedation, akathisia
Clozapine	Suicidal behavior associated with schizophrenia	Oral	Weight gain, sweating, seizures, sedation, risk of diabetes
Felbamate	Severe and refractory epilepsies	Oral	Weight loss, insomnia, dizziness, headache, ataxia, skin rashes, hepatotoxicity
Gabapentin	Partial seizures	Oral	Dizziness, fatigue, hyperactivity and weight gain
Lamotrigine	Partial seizures	Oral, intravenous	Dizziness, headache, diplopia, ataxia, skin rash
Vigabatrin	Partial seizures	Oral	Drowsiness, insomnia, Irritability psychosis, depression, weight gain, blindness
Levetiracetam	Partial seizures	Oral	Leucopenia, fatigue, dizziness, headache, anorexia, psychiatric disturbances
Oxcarbazepine	Partial seizures	Oral	Drowsiness, diplopia, headache, GI distress, hyponatremia, skin rash, Stevens–Johnson syndrome
Phenytoin	Partial seizure	Intravenous	Diplopia, nystagmus, coarsening of facial features gingival hyperplasia, hirsutism, skin rashes, Stevens–Johnson syndrome, agranulocytosis, aplastic anemia, hepatotoxicity
Levodopa	It is used to treat symptoms of Parkinson’s syndrome such as slow movements and stiff, rigid body parts	Oral, intravenous	Nausea, vomiting, and irregular heart rhythms
Sinemet	Treat nausea, vomiting, and irregular heart rhythms, which are side effect associated with levodopa	Oral	Dyskinesias
Pramipexole	It mimic dopamine, thus helping to produce smooth voluntary movement	Oral	Nausea, drowsiness, sleep attacks, hypotension, and hallucinations
Selegiline hydrochloride	It increase the amount of dopamine in the brain and reduces the motor symptoms of Parkinson’s disease	Oral	Nausea, headache, confusion, postural hypotension, hallucinations and insomnia
Tolcapone	It increase the amount of dopamine in the brain and reduce the motor symptoms of Parkinson’s disease	Oral	Dyskinesias, nausea, diarrhea, sleep disturbance, urine dis-coloration and hallucinations
Biperiden hydrochloride	It reduces tremors and muscle rigidity	Oral	Blurred vision, constipation, urinary retention, heat stroke, nervousness, anxiety, confusion, depression, delusions

**Table 2:** Intranasal administration of bioactive agents via in situ gels.

Bioactive Agents	Formulation Composition	Neurological Disorder	Efficacy	References
Levodopa	Pluronic F127, chitosan	Parkinson’s syndrome	Delayed mucociliary clearance	[56]
Amantadine	Pluronic F127, carboxymethylcellulose	Parkinson’s syndrome	No cellular toxicity to human nasal epithelial cells	[57,58]
Ropinirole	Chitosan, hydroxyl propyl methyl cellulose	Parkinson’s syndrome	Enhanced brain uptake of drug in vivo	[59]
Rasagiline mesylate	Poloxamer 407, poloxamer 188, carbopol 934 P and chitosan	Parkinson’s syndrome	6-fold higher drug bioavailability	[60]
Ropinirole	Pluronic F-127 and hydroxy methyl propyl cellulose	Parkinson’s syndrome	Five-fold brain uptake of the drug	[61]
Sumatriptan	Gellan gum	Migraine	High drug concentration in plasma and brain tissues	[62]
Huperzine A	Gellan gum	Alzheimer’s	Increased drug distributions in the rat brain tissues: the cerebrum and hippocampus	[63]
Curcumin	Pluronic F127, Poloxamer	Alzheimer’s	Improved drug-targeting efficiencies in the cerebrum, cerebellum, hippocampus and olfactory bulb	[64]
Geniposide	Poloxamers (P407, P188) and hydroxypropyl methylcellulose	Alzheimer’s	In vitro release profile of the drug was zero-order, and the ex vivo release mechanism was the Weibull model	[65]
Rivastigmine	Poloxamer 407^®^, poly(lactic-*co*-glycolic acid) nanoparticles	Alzheimer’s	Enhanced drug permeability and sustained drug release profile; the cellular uptake of the drug from the formulation was time dependent	[66]
Rivastigmine tartrate	Pluronic F127, HPMC Chitosan, Carbopol 934 and sodium carboxymethyl cellulose	Alzheimer’s	Good distribution to the brain (0.54% ID/g) when compared to intravenous administration	[67]
Doxepin	Chitosan and glycerophosphate, poly(ethylene) glycol	Depression	In vivo studies in Swiss albino mice showed a good increase in activity count and a decrease in immobility time	[68]
Agomelatine	Pluronic F127, Carbopol, chitosan, sodium carboxymethylcellulose, sodium alginate, and hydroxypropyl methylcellulose	Depression	Significantly enhanced brain uptake in vivo	[69]
Fluoxetine hydrochloride	Gellan gum and HPMC (hydroxypropyl methylcellulose)	Depression	Reduced immobility, increased climbing and swimming behavior	[70]
Tramadol hydrochloride	Chitosan, Pluronic, HPMC	Depression	Increased locomotor activity and body weight of the rat model in vivo	[71]
Nortriptyline hydrochloride	Poloxamer 188 and HPMC	Depression	Enhanced drug release profile	[72]
Venlafaxine	Sodium alginate	Depression	Brain uptake was 742.5 ng/mL, t_max_ 60 min; improved swimming and climbing and reduced immobility in vivo	[73]
Paliperidone	Carbopol 934 and hydroxypropyl methyl cellulose	Schizophrenia	High rate of drug permeation via sheep nasal mucosa	[74]
